# Philadelphia County Medical Society

**Published:** 1850-04

**Authors:** 


### PHILADELPHIA COUNTY MEDICAL SOCIETY.

*February* 12, 1850.

President Dr. S. Jackson (late of Northumberland) in the Chair.

The subject of *oil of turpentine* which has been proposed for discussion this evening, was introduced by the President with a few observations which he has since extended and multiplied as follows:

The subject proposed for discussion this evening is, the use of oil of turpentine in typhus fever, (including what has been called the typhoid,) in puerperal and yellow fever, in dysentery, and in hemorrhagies, particularly those that occur in these various diseases.

In all these there would appear to be one general state of the whole system, that of fever with inirritability, requiring more or less stimulation; and happy it is, if physicians have found in turpentine, a stimulus of peculiar operation exactly suited thereto.

The use of this article in these diseases, has been discovered within the present half century, and it now appears to be gaining upon the profession both in Europe and America; hence it may be well for each of us to adduce what he has read and done, thus throwing his mite of latent knowledge into a common stock, for the common benefit of all and for public profit.

The terebinthinate medicines, though much used by our forefathers, esteemed and called by them *the balsam or balm of the viscera,* fell into such neglect that many systematic authors barely mentioned them for a long series of years. Thus the *Dic. des Scien.* Méd., the volume containing this article published in 1821, gives but a meagre account of them and says—"they are much less used than formerly." The *Dic. de* Méd. 2nd edition, vol. 29, published in 1844, has very little to say concerning them, and not one word on their use in hemorrhage or fevers, except a mere passing hint that the oil had been given in puerperal fever. Nor does this comprehensive work advert to them under the head of typhus or of dothinenteritis. Cullen, Eberle, and Paris, speak of the oil in their works on Materia Medica, as a diffusible stimulus, but say nothing of its use in low fevers, dysentery, or hemorrhage; nor do Good, Watson, Gregory, Eberle, McIntosh, Dunglison, name this article in relation to these diseases in their systems of practice, except that Good admits it in chronic dysentery, and Watson merely mentions it for melæna, as a thing he had heard of.

Southwood Smith says nothing of its use in typhus fever, even when there was abdominal pain and flatulence. He once mentions it in hemorrhagic fever:—" now and then he says, a stimulant has a greater effect in checking hemorrhage than an astringent, and then the ol. tereb. is the best remedy." Burne who published an excellent monograph on adynamic fever in 1828, does not name turpentine, though he says much on the tympanitic abdomen, ulcerated bowels, and hemorrhage in this fever. Wilson Philip on fevers, and our own Nathan Smith on typhus, are wholly silent; so is Bartlett, even in his 2nd edition, a work wherein he certainly wished to comprehend all good things. The Library of Pract. Med. gives not a hint concerning its use in hemorrhage, whether active or passive, except in melæna; nor yet in dysentery or fever except the puerperal, and yet Dr. Dunglison has contributed his share, and filled up the supposed deficiencies of the great salmagundi.

Although the utility of oil of turpentine in these diseases, is a late invention, there has been ample time to bring it into more general use; and we can hardly excuse the silence of so many writers on a subject of vital importance with which they ought to have been thoroughly acquainted, for Dr. Chapman in his Therapeutics published in 1817, has written much that goes directly to our present purpose. Now when it is remembered that he had then been lecturing to large classes for four years, the knowledge we refer to, must have been widely diffused.

But Dr. Rush in his history of the yellow fever of Philadelphia in 1805, says that Dr. Physick used it that year with success for the vomiting that occurs in the second stage. It does not appear that he used it in the fully formed black vomit, but merely in that exhausted state of the whole system, and particularly of the stomach, which soon brings on this fatal symptom.

Dr. Brennan, a wild and eccentric physician of Dublin, brought the oil into use in puerperal fever in the year 1814, with undoubted success: his method has since been cried up by some and cried down by others, and as always happens in such cases, without a due regard to the state of the system, and to that peculiarity of action which a remedy may exclusively possess, without our even suspecting it; which, moreover, cannot be ascertained with certainty without a long continued empiricism, scientifically, or at least artfully conducted.

In the London Medical and Physical Journal for 1821, Dr. Copland published a paper on the virtues of turpentine, and this was transferred to volume second of the Journal of Foreign Medical Science, published in Philadelphia, and read beyond the mountains of Pennsylvania in 1822. The author commends it in common dysentery and some hemorrhages, all which is directly to our present purpose; but he details so many other diseases which he cures therewith, as to bring upon himself some slight imputation of quackery. He does not, however, advocate its indiscriminate use in all states of the system. He says—" a phlogistic temperament, though the debility be very considerable, ought to forbid its use. The best general indications for the internal employment of this remedy in hemorrhagic diseases, and those by which I have been guided, are the absence of plethora and the phlogistic diathesis; when there has been a previous great loss of blood; when the pulse is soft, weak and easily compressed, indicating a want of tone in the arterial system. In a word, it can be used in all hemorrhages of a truly passive character." We shall see after a few pages, that he became more bold by practice, and that both he and his New York editor, use the medicine now without regard to the states active and passive.

Dr. Chapman was probably the first to bring the medicine fairly before the American physicians in the treatment of fevers and hemorrhagies; for as said above, he both published and lectured on the subject in the year 1817. In his *Therapeutics* first published this year, he recommends from his own experience, the oil in typhus fevers, when stimuli are called for and in the summer fevers with what he calls a typhoid tendency. He then notices Physic's use of it in the vomiting of yellow fever in 1805. In subsequent editions, I quote from the fifth, he speaks of its great utility in this disease as it stood in 1820, when he and Dr. Hewson gave the oil not merely for the vomiting but as a specific against the whole malady. " Of. the counter agency, says the professor, of turpentine in scalds and burns, we are aware. The stomach in yellow fever, is in a state of inflammation, probably of a somewhat similar nature, which is overcome in the same way. This conjecture derives support from the consideration that, in many instances, the turpentine is soothing in its effects, removing the sense of heat and irritation in that viscus, subduing the force of vascular action and general excitement, and inducing at once a condition of more comfort and security."

He and Dr. Hewson under whose care the hospital at Bush-hill was placed, gave the oil, a drachm every hour, to sixteen patients of whom twelve were cured. " Compared, says the Dr., with what was done in the city by other modes of treatment, this success was exceedingly encouraging. It should too be recollected, that most of the patients were brought into the hospital in an advanced stage of the disease, much reduced by venesection and other evacuations." He then says in a most unfortunate *lapsus ingenii*, that "unless it be employed at the commencement or very early in the attack, the turpentine in common with all remedies, will be for the most part unavailing." Yet twelve out of sixteen cases were cured though in "*the advanced stage and much reduced by venesection and other evacuations*;" to which you may add the agitation of jolting them two or more miles to Bush-hill, after such medical debilitation and that of the disease conjoined. I would rather take the doctor's fact than his opinion, and say that turpentine cured twelve of sixteen deplorable cases.

The professor reasons well on its mode of operation, as far as human reason can go by analogy. But we must take the facts which are sufficient of themselves and there is no need of theorising unless as a means of persuading the incredulous. Drs. Physick and Chapman's facts are incontrovertible, their reasoning may not prove conclusive to every mind, hence we shall not detain the reader therewith.

But, alas for medical experience! Professor Jackson's History of Yellow Fever of 1820, Philada. Med. and Ph. Jour. Vol. II. 11–16, says, the medicine proved less highly beneficial in the city as used by other physicians, and this fact he imputes to the "effects produced by a pure and salubrious air at the hospital, constant medical attention, and good and faithful nursing." All these are good and useful things, but experience has proven, that they will not, when used without turpentine, cure twelve of sixteen such patients as Dr. Chapman describes. Rather than yield my cheering opinions of Dr. Chapman and Hewson's success, I would consider Dr. Jackson's patients or those of whom he speaks, as among the incurables, in consideration of whom he says p. 17, "there is a limit placed beyond which no human effort can extend and where human skill and art cease to avail."

Dr. Charles Caldwell in his history of the yellow fever of 1805, appended to his translation of Alibert, says—"other practitioners derived much advantage in cases of obstinate vomiting, from the use of spirits of turpentine, a remedy first proposed by Dr. Physick. The Dr. appears to have taken the hint in this instance, from the efficacy of that article in preventing gangrene from severe burns. His object was to prevent black vomit and death of the stomach from excessive inflammation. I am sorry to add, that in my hands, that remedy was not productive of those happy effects which are said to have attended its use in the practice of other physicians." Here the Dr. is one against many according to his own account; a strong man certainly, but the battle is not always to the strong. A fortuitous concourse of untoward circumstances, often perverts for a time, the brightest minds in medicine.

Professor Wood in the year 1826, published in the North Amer. Med. and Surg. Jour. vol. i. a paper on the use of the oil in remittent and in typhous fevers. He seems to limit its use to a certain state which appears to be mediate or transitive to either life or death, as nature may adjudge the case, to use an idea of the ancients; for this is their true crisis, when nature sits in judgment, and the patient is ready to go either way. The tongue, after having become moist and begun to clean itself—thus affording indications of a speedy recovery—suddenly becomes dry, smooth, and red; the abdomen becomes tender and distended; the stools dark and offensive; the urine high colored; the skin dry and harsh; the pulse more feeble and frequent; the intellect wanders and the countenance expresses anxiety and suffering.*

Here the professor considers the oil as an almost infallible remedy in a state of things which no other stimulus has been found to relieve. To this proposition we assent most cheerfully. I do not remember having used turpentine in such cases, but I recollect having lost such patients under common stimuli before turpentine came into use. It was a state that I learned to dread, a state on the very confines of death, and alas for the patient if nature or the physician make a single blunder. But *ipsi pauca velim facilem si præbeat aurem:*" I will venture to say to the learned professor, that this perilous crisis must be anticipated and prevented by turpentine. I cannot by any means limit the use of it to the period when the tongue begins to clean, grow smooth, and redden, as a deceptive prelude to the sinking state. While the tongue is yet deeply coated it is often red and dry, the teeth foul and uncovered

* With respect to this delusive cleaning of the tongue, Dr. Rush says, "We sometimes see the tongue after being dry in this fever, suddenly become moist; and yet the patient sinks under the disease."—*Rush's Pringle*, p. 262 note.

—" *dentes retecti*," the whole body with all its functions appearing altogether cachectic—here we may give this medicine without waiting for any tendency in the tongue to throw off its coating, whether this be in typhus or in the protracted state of inflammatory remittent fever, now assuming somewhat of the typhous livery.

A principal duty of the physician is to prevent diseases from running into a state of danger. I can remember myself making a great trepidation about a patient in this very fever, when an old busy-body said—" Poh, Doctor, this woman is in no danger, I have seen worse cases of nervous fever than this." Well, Mr. B. did they recover ? " Oh no, there was Mr. A. B. C.—they all died." I replied, you have said well, they all died, but my wish is to prevent this woman's running into such a state that she too must die.*

I would earnestly enjoin the use of the oil very early in typhus when there is reason, from well known symptoms, to suspect a lesion of Peyer's glands ; for I cannot but hope much from its direct application to the ulcer itself as well as from its reaching it through the blood. And here, in order to be understood, I must say that I consider what has been called *typhoid* as nothing but an accidental state of *typhus*. Both states originate unquestionably from the same contagion or from the same fomes, have the same general symptoms, the same accidental lesions: for what difference is there between an ulcer in Peyer's glands and an ulcer in the brain, the axillary glands, or the suræ, all which are seen in typhns—what difference between one phlegmonous inflammation and another ? This lesion, this effect of disease wherever seated, is red blood in serous vessels, and further than this, no human eyes will ever penetrate. Some careless man in Ireland rolls up his dirty shirt saturated

* Upon looking into Professor Wood's Practice of Med. since the above was printed, I find that he does recommend the turpentine to be used earlier than he proposed in his paper in the North Amer. Med. and Surg. Jour. He says " should the tongue become dry and the abdominal distension be undiminished, the oil of turp. will prove an excellent remedy. I cannot too strongly impress upon the profession the importance of this remedy." And half a page further on, he speaks of it as directly useful in healing the ulcerated ileum. He says too, " it is not improbable that mercury may serve in some degree to arrest the progress of disease in the glands of Peyer."

with perspiration, and after some weeks, he unrolls it in the steerage, thus infecting himself and others, some with typhus and some with the so called typhoid. Now surely this must be one disease, for as Dr. Armstrong says on this very subject—we do not gather grapes from thorns and figs from thistles—when we vaccinate we do not expect a crop of measles: but when we poison ourselves with these fomites, we expect a fever with local inflammation in various parts, which may or may not ulcerate. But I will cease to argue for the reader knows all and even more than I could say in consuming an hour which neither he nor I can spare.*

Upon the contagiousness of this disease when thus generated, I published a paper in the Amer. Jour. of the Med. Scien. for Oct. 1845, which Prof. Wood and Dr. Bartlett have quoted under the head of *typhoid* fever. Call it what you please, it was sometimes the *typhus mitior ;* sometimes the *gravior ;* the febris lenta nervosa and the febris putrida maligna of Huxham from beginning to end and in the same house at the same time. It comes from fomites of our own making, though Drs. Chapman and Bancroft have the grace to believe that it came down from heaven at the beginning of the world, in common with all good things: and Dr. Bartlett says—" our knowledge of its efficient causes, excepting that of contagion, is very limited and imperfect." Now I think the cause is as manifest as that of drunkenness; and I would pledge myself to generate the typhous poison as certainly as a distiller would engage to make a barrel of brandy.

Well then, in what Mr. Morrison of the Newry fever hospital calls the cachectic state of typhus and in the long protracted state

* With respect to the origin of typhous fever, Dr. Rollo relates a fact which may be considered conclusive when taken in conjunction with hundreds of others. Eight men of the horse artillery were taken with a suspicious fever, and upon inquiry it was found, that their rooms had entirely different bedding from the rest of the barracks. The hammocks were rolled up tightly every morning the moment the men rose, and were not opened till about to be occupied at night; nor had they been aired for more than two months, owing to a succession of wet weather; and therefore upon opening them for examination a very nauseating smell was perceived. "Here, says Dr. Rollo, an infectious fever evidently arose from the confinement of the effluvia of a man's own person, in a term of about two months." See Hygeia xi. 45. Much to the same purpose may be found, but particularly in Willan's Reports p. 255.

of koinomiasmatic fever, I must from much experience maintain, that the oil of turpentine is a remedy of inestimable value, and without an equal. Where it was that I first learned the use of this article in the diseases above referred to, I cannot recollect, but I clearly remember having used it internally in cholera morbus in 1823, and soon after in typhus fevers, with such success, that it soon became with me a favorite remedy.

In cholera morbus, when the system appeared to be in a sinking state, after the puking and purging had been subdued, pulse very feeble, some fever, coldness of the extremities, listlessness, tendency to stupor—here this oil in doses of half a dram to a dram every hour, appeared to be a very important remedy. Whether I learned this from books, I do not know, for of the farrago of knowledge now in my head like lumber in a garret, I cannot render unto every Cæsar the things that are his.

With respect to the use of the oil in typhus fever when there is meteorism, diarrhœa, and therefore a probable inflammation of Peyer's glands, it affords a hope which no other stimulus does. And here I cannot but remark, as I did some years ago in the Transactions of our College, that although the indications of these ulcers now existing or impending, appear to be almost infallible, nothing specific is done to prevent or to cure them. They are not an inconsiderable item in the history of the disease; for who knows how soon they may bore through the bowels, so that a patient who appears to be safe, is really on the verge of a mortal symptom? Let us be on the alert and say with Cato—"*vigilando, agendo, bene consulendo, prospere omnia cedunt.*" Hence the present writer suggested to the College in his Report on the Theory and Practice, *Transactions vol. I.* 353, that something ought to be done specifically for these dangerous ulcers and says—" blisters have been found useful excitants and revellents in this disease: we have found them highly beneficial in cases attended by hemorrhagic diarrhœa, would they not if repeatedly applied to the abdomen, derive inflammation from the ileum, particularly if preceded by cups? The mortality of this lesion surely requires a specific interference; we propose then, calomel or hydriodate of potash, as used by Mr. Morrison; sp. terebinthinæ; cups and frequent blisters to the abdomen."

When calomel is given in very small, frequent doses, as half a grain every three hours, it may be managed so as to cure the local disease without a salivation which is a horrible thing. The hydriodate of potash possibly acts on the local phlogosis specifically, as it was found so highly beneficial by Mr. Morrison. Cups to the abdomem followed by a blister and this kept discharging pus by means of savin or mezereon cerate, surely promises much in theory. *Pars dolens trahit*—there must be a determination of morbid action from the ileum to the skin, when a steady and copious efflux of humours is there maintained. Nay, not merely from the inflamed bowel will the attraction be established, but from the whole diseased body. *Ubi est irritatio ibi fluxus*, is an old motto in such cases; but as there is no pain, no irritation in a blister thus running with pus, I would rather have a motto more suited to the fact and say, *ubi est fluxus ibi morbus exit.* Or you may say of the disease as Cicero did of Catiline, only changing the tense, *abit*, *excedit*, *evadit*, *erumpit.*

With respect to the hydriodate of potash, I will say briefly, that I was lately called to assist Dr. Neville C. Reid in a case of the said typhoid fever—such a case as Stahl's expectation would not cure. I found the Dr. using the hydriodate in doses of 3 grs. every two hours, with little else except a well regulated diet. I encouraged him to go on with the medicine; the fever gradually wore away, and the patient was restored to sound health. Dr. Reid tells me that he has used this medicine in several cases with entire satisfaction.

In the cachectic state of dysentery the very same advantages are obtained from turpentine; but here a very different state of things obtains. In idiopathic fevers, there is something to contend with beside the inflammation, and therefore this stimulus cannot be used till the phlogistic diathesis be much on the wane; but in dysentery, the local disease is paramount; and since this is concentrated in a mucous membrane and has no tendency to a translation, and more particularly since the oil seems peculiarly suited to inflammation of this very organ, it may be given rather early in the disease. Therefore, though its energy appears most conspicuous in the cachectic state, it will be found eminently useful in preventing this state, if given early with all the necessary co-adjuvants.

Nor is it to be neglected as an external remedy. When diligently rubbed on the abdomen, it is soon perceived in the breath and the urine; and of course it is carried to the diseased membrane. I have thus profited much by it in cases where leeches and blisters were not thought requisite.

In bringing this subject before the Society, my intention was, to limit the discussion to its use in that flaccid state of the system, and particularly of the mucous membrane, in which there is a great want of excitability and sometimes and in some diseases, a near approach to a total extinction thereof. This is seen in the low stages of typhus and remittent fevers, in the same stage of yellow fever, plague, puerperal fever, and in the hemorrhage which often attends them, particularly from the bowels. We do not wait for this low state in dysentery because the general fever is fast passing off by the bowels, and the undue stimulus of the turpentine may be carried away therewith.

In the hemorrhage which often attends bilious remittent and typhus fevers and in melæna, I have had such success as leads me to consider it an almost indispensable remedy. I certainly did not rely upon it in bad cases to the exclusion of other potent means, such as ice, blisters, astringents; but by trusting to it in the milder cases and watching its supposed effects in the more severe, I have carefully formed of it a most favorable opinion. In hæmoptysis, epistaxis, wounds, and other active hemorrhagies, I have never tried it, but I have been called to cases of all these forms in which the patients had been using it with apparent advantage.

Dr. Copland in his Dict. appears to recommend it in all hemorrhagies active, passive, and neutre. He says, " it constringes the capillaries of the part to which it is applied," and " when absorbed into the circulation, its astringent effects on the capillaries is very remarkable." " When the dose is large, it reduces the frequency and strength of the heart's action, especially when they are much increased, hence it is an appropriate remedy in the more active forms of hemorrhage, inasmuch as with its astringent action on the capillaries, it weakens the *vis à tergo*. " When given in smaller doses and carried into the blood, it increases the tone and changes or modifies the action of the extreme vessels." " The existence of inflammatory action does not contraindicate its use, for it lowers vascular excitement and prevents effusion and the formation of coagulable lymph, especially when taken in sufficiently large and repeated doses." He cautions however against large doses when the powers of life are much depressed and when the patient has lost much blood.

Dr. Lee, the New York editor of Copland, says, "we have found turpentine one of the most valuable remedies in every form of hemorrhage. It is rapidly taken into the circulation, as manifested by its odour in the urine and breath, and it exercises plainly an astringent effect on the capillaries. It posesses moreover the important effect of operating more promptly than any other astringent, as we have often observed in hemoptysis which had resisted other remedies. With proper precautions it may be given both in the active and passive forms of the disease."

After such commendations as Copland and Lee's, one might suppose there was nothing more to be desired, and yet the praise is not more extravagant than that of John Hunter. He applied it to divided arteries and gave it internally in active hemorrhage as epistaxis and said, "it was the best if not the only styptic." See Braithwaite part xix.—72.

Dr. Chapman commends the oil in hemetemesis, melæna, and in passive hemoptysis; then, as though to arrest the attention of the reader and make upon him an impression not to be forgotten, he turns his beautiful English wrong end foremost and thunders out in Ciceronian diction—"equal proof have I of its use in hemorrhoids, alike restraining the flow of blood and soothing irritation." After such a commendation from such a man, who could be so criminal as to neglect the experiment? Surely when this oil is attainable, no one ought to prescribe sugar of lead which I know from experience does sometimes bring on its own peculiar colic, even when given with acetic acid.

*Multa desunt:* but I have fulfilled in a measure my first proposition—that turpentine was for a long time neglected, and that when brought into use by some, it was too much neglected by others; and finally I have advanced some authorities for its use, which ought to lead on to further inquiries. The reader will probably say of the present writer as Johnson says of a great poet —"Pope in the chair of wisdom tells us much that every man knows and much that he does not know himself."

